# Sex differences in ventricular–vascular coupling following endurance training

**DOI:** 10.1007/s00421-014-2981-z

**Published:** 2014-08-21

**Authors:** A. D. Lane, H. Yan, S. M. Ranadive, R. M. Kappus, P. Sun, M. D. Cook, I. Harvey, J. Woods, K. Wilund, B. Fernhall

**Affiliations:** 1 Department of Health and Human Physiology, University of Iowa, Iowa City, IA USA; 2 Department of Kinesiology and Community Health, University of Illinois at Urbana–Champaign, Champaign, IL USA; 3 Department of Anesthesiology, Mayo Clinic, Rochester, MN USA; 4 Department of Kinesiology, Nutrition, and Rehabilitation, University of Illinois at Chicago, Chicago, IL USA; 5 Physical Activity and Health, East China Normal University, Shanghai, China; 6 School of Family Studies, University of Connecticut, Storrs, CT USA

**Keywords:** Exercise, Systolic performance, End-systolic pressure

## Abstract

**Introduction:**

Ventricular and vascular coupling is defined as the ratio of arterial elastance (Ea) to ventricular elastance (Elv) and describes the interaction between the heart and arterial system. There are sex differences in both arterial and ventricular function in response to both acute exercise and aerobic exercise training.

**Purpose:**

To examine the effects of aerobic exercise training on elastances and the coupling ratio in young adult men and women. We hypothesized a reduction in the coupling ratio in both sexes due to a decrease in Ea that would be more pronounced in men and an increase in Elv that would be larger in women.

**Methods:**

Fifty-three healthy, young adults completed the study. Central pulse wave velocity and heart volumes were measured before and after an 8-week aerobic training intervention. Elastances were calculated as Ea = end-systolic pressure/stroke volume and Elv = end-systolic pressure/end-systolic volume and indexed to body surface area.

**Results:**

After the intervention, women augmented indexed and un-indexed Elv from 2.09 ± 0.61 to 2.52 ± 0.80 mmHg/ml, *p* < 0.05, and reduced the coupling ratio from 0.72 ± 18 to 0.62 ± 15, *p* < 0.05, while men maintained their pre-training ratio (from 0.66 ± 0.20 to 0.74 ± 0.21, *p* > 0.05). Women also reduced end-systolic pressure (from 91 ± 10 to 87 ± 10 mmHg), and both groups reduced central pulse wave velocity (from 6.0 ± 1.0 to 5.6 ± 0.6 m/s, *p* < 0.05).

**Conclusion:**

We conclude that after 8 weeks of aerobic training, only women reduced their coupling ratio due to an increase in Elv. This suggests that aerobic exercise training elicits sex-dependent changes in the coupling ratio in young, healthy individuals.

## Introduction

Ventricular–vascular coupling can be expressed as the ratio of arterial elastance (Ea) to left ventricular elastance (Elv; Ea/Elv) (Sunagawa et al. 1983). Ea quantifies the workload imposed on the ventricle by the arteries (a measure of afterload) and Elv is a load-independent measure of cardiac performance. The coupling ratio characterizes the interplay between the heart and arterial system in the same time domain (Chantler et al. 2008) and describes how the left ventricle and the arterial system impact each other to modify cardiovascular reserve, cardiac performance and peripheral hemodynamics (Chantler et al. 2008).

Increased Ea/Elv not only can be structurally attributed to stiffer arteries and reduced cardiac compliance but may also indicate functional capabilities of the heart or arteries (Chantler et al. 2008). Importantly, the coupling ratio has been linked to long-term survival in clinical cohorts, including patients recovering from myocardial infarction (Antonini-Canterin et al. 2009).

There are known sex differences in arterial properties; young women have reduced small vessel compliance and increased augmentation index (AIx) compared to men despite having lower systolic blood pressure and pulse pressure (Winer et al. 2001). This may result in divergent Ea responses to aerobic training despite similar increases in large artery compliance following training as Ea comprises both pulsatile and steady types of arterial resistance (Chemla et al. 2003).

There are also sex differences in resting left ventricular function, with healthy women exhibiting increased Elv and diastolic stiffness compared to men, as well as having significantly higher amounts of wasted left ventricular effort (Hayward et al. 2001; Lane et al. 2013). In fact, women tend to have higher Ea and Elv than men throughout life (Najjar et al. 2004; Redfield et al. 2005), adding further support for the notion that sex differences may occur following training. Despite these differences, coupling ratios are similar in men and women, reflecting the ability of the heart and vasculature to adapt in tandem to maintain efficiency and cardiac output (Najjar et al. 2004). Acute exercise causes an increase in Ea and Elv in both sexes (Najjar et al. 2004), but women have been shown to develop greater myocardial wall stress at any given arterial load or ventricular geometry (Chirinos et al. 2012), and the repetitive, acute challenges associated with exercise training may exacerbate these differences.

Resting Ea/Elv is reduced following aerobic exercise training in men with coronary artery disease, due to a decrease in Ea following training, and these patients demonstrate enhanced Elv during afterload stress (Rinder et al. 1999). In contrast, older women may not improve ventricular performance with exercise training (Spina et al. 1993). To date, the only study that examined the effects of exercise training specifically on ventricular and arterial elastances (Shibata et al. 2011) showed that older women were unable to improve the coupling ratio. However, age at the onset of exercise training may influence the ability to improve coupling, because both male and female master’s athletes maintained pressure–volume relationships similar to younger controls while sedentary older adults had altered pressure–volume relationships (Fujimoto et al. 2012; Arbab-Zadeh et al. 2004). Because age at the onset of exercise training may affect the training response, it is possible that aerobic exercise training may result in different Elv responses in younger women, and there may be a sex-specific role of age at exercise onset in disease prevention, i.e., a “window of opportunity” in which women may improve ventricular performance in a similar manner as young and older men.

The purpose of this investigation was to determine how sex affects Ea/Elv following 8 weeks of aerobic training in healthy, previously sedentary young men and women. We hypothesized that men would reduce Ea to a greater extent than women, but that women would augment Elv to a greater extent than men and that this would result in an attenuated Ea/Elv following the training intervention in both sexes.

## Methods

### Subjects

A total of 53 (*M* = 28; *F* = 25) nonsmoking, normotensive (resting blood pressure <140/90 mmHg), sedentary subjects between 18 and 35 years participated in this study. Subjects were classified as sedentary based on their exercise habits for the past 6 months (no structured exercise of any kind lasting >30 min >1 time per week). None of the subjects were taking chronic medications except oral contraceptives; none had been diagnosed with any chronic disease. They were instructed to refrain from non-prescription medications, i.e., nonsteroidal anti-inflammatories, aspirin, and naproxen, for the week preceding testing. All subjects signed informed consent and the study complied with the Declaration of Helinski and was approved by the University of Illinois at Urbana–Champaign institutional review board.

### Study design

Subjects reported to the laboratory for one visit before and after an 8-week endurance exercise training program. Women were tested in the early follicular phase (or placebo phase of oral contraceptives). Subjects were instructed to be 4 h postprandial and to abstain from caffeine and alcohol for at least 12 h before testing, and had refrained from heavy physical activity in the 24 h before testing. Subjects had previously completed a physical activity and health history questionnaire to confirm sedentary status and ensure safe participation in maximal exercise testing. Measurements of height, weight and blood pressure (BP) were obtained. Pulse wave analysis measurements were made and cardiac ultrasonography was performed using a high-fidelity ultrasound. Next, the subjects underwent a VO_2peak_ test. Subjects then began an endurance exercise intervention, exercising 3 times per week at 60–90 % of maximal heart rate (HR) for 30–60 min. A final testing session was conducted after 8 weeks of training, as training for 4–12 weeks has been shown to induce vascular adaptation in similar populations (Beck et al. 2013; Collier 2008), also in the early follicular phase/placebo phase of oral contraceptives in female subjects. All subjects were required to have completed a minimum of 21 of the 24 monitored, scheduled sessions (87 % completion) prior to their final cardiovascular testing session.

### Anthropometrics

Standing height and weight measurements were taken with subjects wearing light-weight clothing using a stadiometer and balance-beam scale. Body surface area (BSA) was calculated using Mosteller’s formula (1987).

### Brachial artery BP assessment

Resting systolic BP (SBP) and diastolic BP (DBP) were measured at the brachial artery using an automated oscillometric cuff (HEM-907 XL; Omron, Shimane, Japan) after at least 5 min of supine rest in a quiet, dimly lit room. Brachial BP was obtained in duplicate with 1 min between the first and second reading. If the two values were not within 5 mmHg, another measurement was taken until 2 values within 5 mmHg of each other were obtained. Values within 5 mmHg of each other were averaged and used for analysis.

### Pulse contour analysis

The aortic waveform was reconstructed using radial artery pressure waveforms obtained in the supine position from a 10-s epoch using applanation tonometry (Millar Instruments, Houston, TX) and calibrated using brachial systolic and diastolic BP (Sharman et al. 2006). Using a generalized validated transfer function (Sharman et al. 2006), a central aortic pressure waveform was reconstructed from the radial artery pressure waveform (SphygmoCor; AtCor Medical, Sydney, Australia) to obtain central BP, augmentation index (AIx), and end-systolic pressure. Aortic mean arterial pressure was determined from the integration of the reconstructed aortic pressure waveform using the SphygmoCor software. An in-device quality rating of ≥80 % was required for all recordings used in analysis.

### Pulse wave velocities

Pulse wave velocities (PWV) were measured using previously described techniques (Van Bortel et al. 2002). Distances from the suprasternal notch to the femoral artery and from the carotid artery to the suprasternal notch were measured as straight lines with a tape measure and recorded to the nearest mm. The distance from the carotid artery to the suprasternal notch was then subtracted from the distance between the suprasternal notch and femoral artery to account for differences in the direction of pulse wave propagation. For distal PWV (dPWV), the distance was measured in a straight line with a tape measure between the femoral artery and the dorsalis-pedis artery and recorded to the nearest mm. Using the same high-fidelity strain-gauge transducer as in the pulse contour analysis measurements, pressure waveforms were taken at the right common carotid artery and then at the right femoral artery for central PWV (cPWV). Waveforms were obtained from the right femoral artery and then at the right dorsalis-pedis artery for distal PWV. PWV was calculated from the distances between measurement points and the measured time delay between 10 proximal and distal waveforms (SphygmoCor; AtCor Medical). The peak of the R wave recorded from the ECG was used as a timing marker. Due to the influence of blood pressure on PWV, we also calculated cPWV controlled for aMAP (cPWV/aMAP).

### Cardiac echocardiography

Cardiac output (CO) and volumes were assessed at rest using two-dimensional echocardiography (Aloka Alpha-10, Tokyo, Japan). With subjects in the left lateral position, measurements were obtained using the four-chamber apical view. The interior endocardial border of the left ventricle was traced manually during both end systole and end diastole. Volumes were measured using Simpson’s rule. Stroke volume (SV) was calculated by subtracting end-diastolic volume (EDV) from end-systolic volume (ESV). CO was calculated as HR multiplied by SV. Ejection fraction (EF) was calculated from the ventricular volumes and expressed as a percentage of SV to EDV. Systolic (Sʹ) and early diastolic (Eʹ) tissue velocities were derived from tissue Doppler imaging with the cursor placed at the lateral mitral annulus. Left ventricular mass (LVM) was determined using the Teicholz formula and M-mode echocardiography. Three beats were measured and the average of the measurement was used in analysis.

### VO_2peak_ test

Subjects began with a warm-up period, consisting of pedaling on an upright cycle ergometer (Lode Excalibur, the Netherlands) at 60–100 rpm at 30 W for 30 s. They then started the test by pedaling at 50 W for 2 min. Every 2 min thereafter, workload was increased by 30 W until test termination. Heart rate was measured with a Polar heart rate monitor (Polar Electro, Woodbury, NY). Expired air was analyzed with a Quark b^2^ breath-by-breath metabolic system (Cosmed, Rome, Italy). The test was terminated when subjects could no longer continue, and maximal effort was determined based on meeting three of the following five criteria: (1) a final rating of perceived exertion score of ≥17 on the Borg scale (scale 6–20), (2) a respiratory exchange ratio >1.1, (3) plateau in HR with increased workload, (4) a “plateau” (increase of no more than 150 ml) in oxygen uptake with an increase in workload, (5) volitional fatigue, i.e., an inability to maintain a pedal rate above 60 rpm.

### Calculation of Ea/Elv

Arterial elastance was calculated using the equation Ea = ESP/SV and ventricular elastance was calculated as Elv = ESP/ESV (Chantler et al. 2008). ESP was derived from the arterial waveform as this method of obtaining ESP is more reliable than using a calculation (Kappus et al. 2013). Both Ea and Elv were then indexed to body size by dividing by BSA due to the documented effect of body size on these variables (Chirinos et al. 2009). The ratio of Ea/Elv and this measure indexed to body size (EaI/ElvI) were both used for analysis, and both un-indexed and indexed values are presented in the results section.

### Calculation of wasted left ventricular effort (*E*_w_)

Wasted left ventricular effort was calculated using the equation *E*_w_ = 2.09 × AG × (ED−Tr) (Hashimoto et al. 2008). In this equation, AG is augmented pressure, or the difference between systolic pressure and the peak pressure generated by initial pressure as well as the contribution made by reflection of the pulse wave. ED is the entire ejection duration, Tr is the systolic travel time of the propagated wave, and 2.09 is a constant that is used as a surrogate for one-half the area of an ellipse, the approximate shape of the wasted pressure portion of the reconstructed aortic waveform (Hashimoto et al. 2008).

### Exercise training intervention

Study subjects were permitted to select their own mode of exercise from among commonly available cardiovascular equipments (upright cycle, recumbent cycle, elliptical trainer, and treadmill), but were required to exercise a minimum of 30 min per week using an upright cycle ergometer to prepare for the final VO_2peak_ test. We believe that this protocol makes our findings more representative of training that would be seen in a “real-life” situation when previously sedentary individuals embark upon cardiovascular training. Heart rate was taken every 10 min during the exercise session using a commercially available HR monitor (Polar, Electro, Woodbury, NY). If HR was below 65 % of previously determined maximum heart rate, participants were asked to increase speed or resistance to achieve appropriate HR. If the early follicular phase of menstrual cycle did not precisely coincide with the 8-week training mark, subjects exercised until the onset of this phase to control the effects of hormonal fluctuations on our measurements.

### Repeatability of measures

All data were analyzed by a single reviewer, and our coefficient of variability was 8.7 % for EDV, 4.7 % for ESV, and 14.3 % for SV. In a smaller subset of participants (*n* = 11), intraclass correlations (ICC) were calculated to assure repeatability of variables. After a 4-week control period before exercise intervention, we repeated blood pressure, arterial stiffness, cardiac measures and elastance calculations as previously described. We obtained an ICC of 0.85 and 0.95, respectively, for arterial and ventricular elastances, indicating good reliability of our measurements.

### Statistical analysis

Sample size was determined using data from relevant studies examining the effect of exercise training on Ea/Elv (34). With a β of 80 % and an alpha level of 0.05, we determined that 21 men and women (42 total participants) were needed to test our hypothesis. Normality of variables of interest was assessed using Shapiro–Wilk tests. Non-normally distributed variables were log transformed before further analysis. A repeated measures, 2 × 2 ANOVA was performed to test for differences between sex and time points in variables of interest. Results shown are mean ± SD. Significance was declared if *p* < 0.05. Statistical Software for the Social Sciences (SPSS, Chicago, IL) version 17.0 was used.

## Results

### Subject characteristics

The mean age for both sexes = 24 ± 1 years. Weight and BMI did not change in either sex after training (*p* > 0.05). Men had higher VO_2peak_ than women (*p* < 0.05), but both groups increased VO_2peak_ (by 13 % for both men and women) after training (*p* < 0.05) without an interaction effect; Table 1.

**Table 1:** Hemodynamic changes after 8 weeks of endurance training by sex (mean ± SD)

	Male pre-training	Male post-training	Female pre-training	Female post-training	*p* value for interaction
Weight (kg)^‡^	81 ± 10	81 ± 10	65 ± 12	65 ± 12	0.634
HR (bpm)	64 ± 10	63 ± 8	64 ± 8	65 ± 9	0.635
BSA (m^2^)	2.03 ± 0.16	2.02 ± 0.17	1.81 ± 0.35	1.81 ± 0.35	0.277
BMI (kg/m^2^)	25 ± 3	25 ± 3	25 ± 4	25 ± 4	0.248
VO_2peak_ (ml O_2_/kg/min)*^‡^	38 ± 8	43 ± 6	30 ± 5	34 ± 4	0.804
SBP (mmHg)^‡^	128 ± 8	124 ± 12	116 ± 8	111 ± 9	0.974
DBP (mmHg)	70 ± 9	69 ± 7	70 ± 10	68 ± 10	0.771
MAP (mmHg)^‡^	89 ± 9	88 ± 8	86 ± 9	83 ± 9	0.062
PP (mmHg)^‡^	60 ± 9	58 ± 8	46 ± 9	44 ± 8	0.778
aSBP (mmHg)^‡^	106 ± 8	104 ± 10	102 ± 10	98 ± 10	0.586
aDBP (mmHg)	71 ± 10	70 ± 10	72 ± 8	69 ± 11	0.506
aMAP (mmHg)	87 ± 8	85 ± 9	86 ± 10	82 ± 10	0.500
aPP (mmHg)^‡^	35 ± 5	34 ± 5	30 ± 5	29 ± 5	0.859
ESP (mmHg)^†^	91 ± 8	91 ± 10	91 ± 10	87 ± 10*	0.045
AIx (%)^‡^	−1 ± 10	0 ± 12	12 ± 10	12 ± 11	0.771
cPWV (m/s)*^‡^	6.2 ± 01.0	5.8 ± 0.6	5.7 ± 1.2	5.3 ± 0.7	0.844
dPWV (m/s)	8.6 ± 1.8	8.3 ± 1.6	8.2 ± 1.5	8.3 ± 1.8	0.905

*SBP* sytolic blood pressure, *DBP* diastolic blood pressure, *MAP* mean arterial pressure, *PP* pulse pressure, *aSBP* aortic systolic pressure, *aDBP* aortic diastolic pressure, *aMAP* aortic mean arterial pressure, *aPP* aortic pulse pressure, *ESP* end-systolic pressure, *AIx* augmentation index, *cPWV* central pulse wave velocity *dPWV* distal pulse wave velocity

*p* < 0.05 for all

* A significant difference between pre- and post-training values in the whole cohort

^†^A significant time by sex interaction

^‡^A significant difference between male and female values, both pre- and post-training

### Blood pressures

Pre- and post-training blood pressures and central hemodynamic variables are presented in Table 1. There were no interaction effects for brachial or aortic BP. ESP was reduced in women but not in men (interaction *p* < 0.05). There were no changes in AIx in either sex, although there were significant differences by sex (*p* < 0.05) that were unaffected by training. cPWV decreased (*p* < 0.05) in the whole cohort post-training; Table 1. When we controlled for pressure (cPWV/aMAP), this reduction was no longer significant (*p* = 0.26 for main effect of training), from 0.067 ± 0.010 to 0.068 ± 0.010 in men and from 0.071 ± 0.010 to 0.067 ± 0.005 in women.

### Echocardiography

Men and women had similar SVI and ESVI and these variables did not change with training, Table 2. Men had larger EDVI pre- and post-training, *p* < 0.05, but these values were unchanged by training in both sexes, *p* > 0.05. Ejection fraction was maintained with training in men but increased in women (*p* < 0.05; Table 2). Two extreme outliers (both male) were removed before further analysis of Sʹ velocity. Sʹ velocity was increased in women but not in men following training (*p* < 0.05 for interaction effect), but Eʹ was maintained with training in both sexes, *p* > 0.05 for time and interaction effects, Table 2. LVM indexed to BSA (LVMI) was higher in men both pre- and post-training, but there were no changes with training, nor was there an interaction effect of sex for LVMI (*p* > 0.05; Table 2).

**Table 2:** Cardiac variables before and after training by sex (mean ± SD)

	Male pre-training	Male post-training	Female pre-training	Female post-training	*p* value for interaction
LVMI (g/m^2^)^‡^	122 ± 33	118 ± 37	79 ± 22	79 ± 21	0.408
SVI (ml/m^2^)	37 ± 13	37 ± 2	36 ± 10	35 ± 8	0.963
ESVI (ml/m^2^)	24 ± 10	26 ± 9	25 ± 8	22 ± 7	0.055
EDVI (ml/m^2^)^‡^	69 ± 13	65 ± 13	62 ± 13	57 ± 13	0.062
EF (%)^†^	61 ± 7	59 ± 6	59 ± 6	62 ± 7*	0.025
Sʹ (cm/s)^†^	11.1 ± 3	10.8 ± 2	10.8 ± 2	12.3 ± 3*	0.026
Eʹ (cm/s)	17.7 ± 4	17.9 ± 4	18.5 ± 4	19.7 ± 4	0.405
*E*_w_ (dyne cm^−2^ s)^‡^	94 ± 271	7 ± 318	1,506 ± 281	1,599 ± 258	0.623

*LVMI* left ventricular mass indexed to body size, *SVI* indexed stroke volume, *ESVI* indexed end-systolic volume, *EDVI* indexed end-diastolic volume, *EF* ejection fraction, *S*ʹ early systolic myocardial velocity, *E*ʹ early diastolic myocardial velocity, ∆*E*_w_ wasted left ventricular effort

*p* < 0.05

* A difference between pre and post-training values in the whole cohort

^† ^A time by sex interaction

^‡ ^A significant difference between sexes at both time points

### EaI

**Fig. 1:**
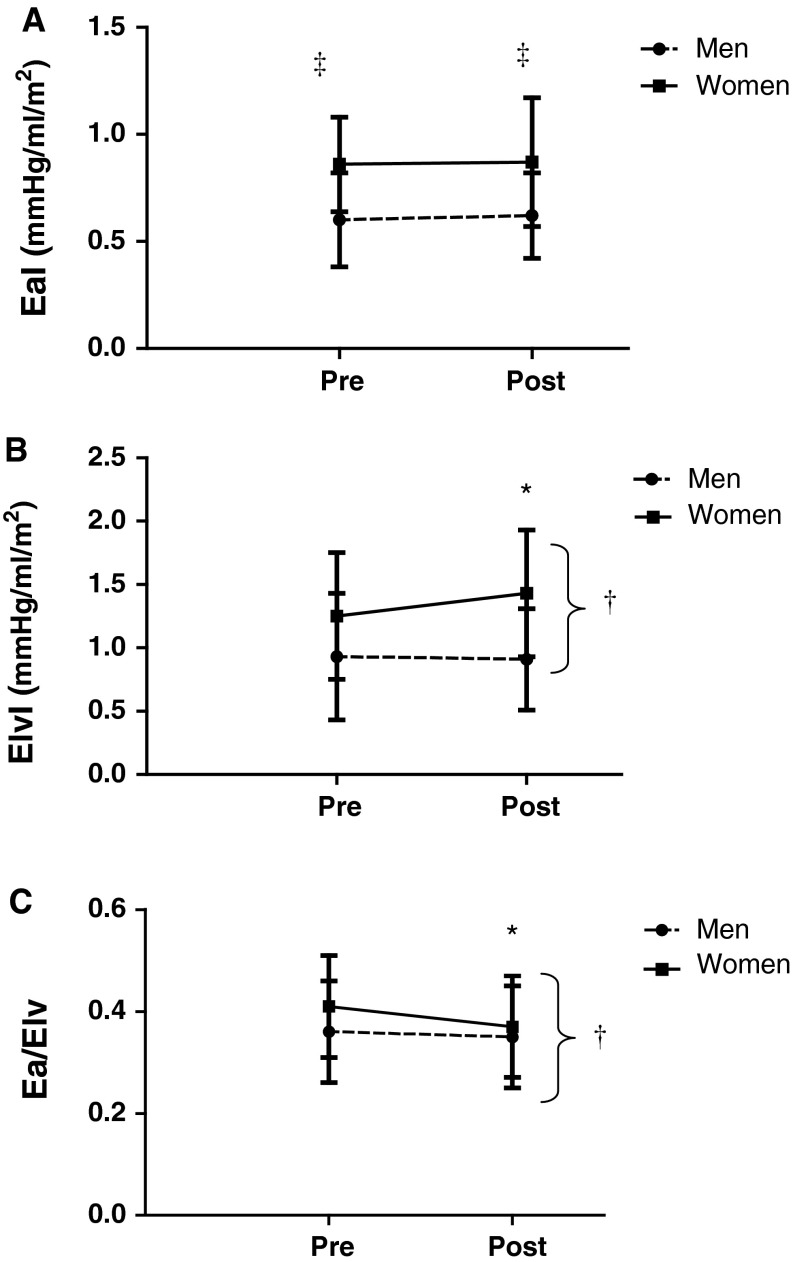
**a** EaI, **b** ElvI, and **c** ratio responses to exercise training in men and women. *EaI* arterial elastance indexed to body size; *ElvI* left ventricular elastance indexed to body size; *EaI*/*ElvI* the coupling ratio indexed to body size; *asterisk* represents a significant difference between pre- and post-training values in that sex; *dagger* indicates a significant time by sex interaction; and *double dagger* denotes a significant difference between male and female values at that time point, *p* < 0.05 for all. Men and women maintained EaI post-training. Female subjects increased ElvI and decreased Ea/Elv after training. EaI, ElvI, and ratio responses to exercise training in males and females

At baseline and after training, women had higher EaI than men, but EaI did not change with training in either group, *p* > 0.05; Fig. 1a. Un-indexed values of Ea were also not altered with training (from 1.48 ± 0.7 to 1.47 ± 0.08 mmHg/ml in women and from 1.22 ± 0.06 to 1.27 ± 0.06 mmHg/ml in men, *p* > 0.05).

### ElvI

At baseline, women had higher ElvI than men (*p* < 0.05) and only women increased ElvI following training (*p* < 0.05; Fig. 1b). The un-indexed Elv values responded similarly (from 2.09 ± 0.61 to 2.52 ± 0.80 mmHg/ml in women and from 1.96 ± 0.86 to 1.90 ± 0.70 mmHg/ml in men; *p* < 0.05).

### Ea/Elv

The coupling ratio was attenuated in women but unchanged in men following training (*p* < 0.05; Fig. 1c). This effect persisted when un-indexed values were considered (from 0.72 ± 18 to 0.62 ± 15 mmHg/ml in women and from 0.66 ± 0.20 to 0.74 ± 0.21 mmHg/ml in men, *p* < 0.05 for interaction).

### Wasted left ventricular effort

Women had higher *E*_w_ than men (*p* < 0.05), but this measure was not altered with training, Table 2.

## Discussion

The main finding of this study was that young men and women have different ventricular elastance responses to 8 weeks of endurance exercise training. The augmentation of Elv in young women resulted in a reduction in the coupling ratio and increase in EF, while men retained their pre-training values. These effects of training persisted even when indexed values were evaluated, thus body size per se did not appear to influence the findings. The significantly higher Ea and EaI in women both pre- and post-training may have important implications for long-term cardiovascular health as augmented Ea indicates increased ventricular afterload and has been associated with increased left ventricular mass and decreased systolic performance (Saba et al. 1999). Interestingly, despite reductions in cPWV, EaI was not altered in either men or women, suggesting that central arterial stiffness on its own may not be associated with ventricular afterload in this young, healthy population. Ea represents both pulsatile and steady components of resistance, but it is 3 times more sensitive to the resistance-to-HR ratio versus compliance (PWV) component (Chemla et al. 2003; Segers et al. 2002). As HR and AIx were maintained post-training, this may account for the maintenance of Ea (despite a pressure-driven reduction in PWV, i.e., systemic compliance). The fact that ventricular volumes themselves were not significantly altered after short-term training was not surprising as this has been previously reported (Sipola et al. 2009). Because neither EDV nor LVM was significantly altered with training in either sex, structural changes are unlikely to be the cause of the functional improvements (EF, Elv) observed in female participants. It is possible that a lengthier training stimulus may cause structural changes that further influence elastances, but this idea warrants investigation in future studies.

To our knowledge, this is the first study that examined the effect of exercise training on elastances in young, healthy men and women. The increase in Elv and ElvI in young healthy women may be due to different mechanisms than those associated with aging, particularly because ESP (which is directly related to Elv) was reduced in our cohort. In contrast, older women experience a concomitant rise in ESP and Ea that also causes augmentation of Elv, potentially resulting in left ventricular hypertrophy (LVH) (Kass and Kelly 1992). The reduction in ESP and cPWV and increase in S′ velocity in this study suggest that other factors, such as intrinsic myocyte contractility or improvement in calcium handling (Locatelli et al. 2013), contributed to the increased contractile performance and augmented ventricular elastance in young women. This is further supported by the finding that the reduction in the coupling ratio occurred concomitantly with an augmentation in EF, consistent with the notion that the coupling ratio is inversely related to EF (Sunagawa et al. 1983).

It is also unlikely that augmentation of ElvI in young women occurred to offset an increase in central arterial load, as is the case in sedentary aging (Najjar et al. 2004). This distinction between enhanced contractility and augmented ventricular stiffness is important when considering development of pathology, as the progression towards LVH is caused by increased arterial resistance and characterized by uncontrolled high blood pressure and heightened arterial stiffness (Drazner 2011). Thus, it is likely that the augmentation of ElvI post-training in our cohort represents a beneficial increase in myocardial performance, primarily caused by improved contractility (as suggested by the increase in S′ velocity along with no change in E′ velocity, a relatively load-independent indicator of ventricular relaxation). This is in contrast to the increase in Elv older or hypertensive individuals (Redfield et al. 2005), where chronically augmented Elv in older adults or hypertensive patients is due to stiffening of the ventricle and arteries (Kass and Kelly 1992). Chantler et al. also urged caution in interpreting ventricular elastance changes in younger men and women as detrimental to net cardiovascular performance (Chantler et al. 2008). The increase in ElvI in our study does *not* appear to be linked to a reduction in function or increased stiffness of the heart and arteries. Rather, it probably reflects a desirable increase in performance, as evidenced by a tandem increase in Elv, S′, and EF, maintenance of E′, and reduction in ESP (Chantler et al. 2008; Sunagawa et al. 1983).

Prior studies have characterized the development of specific types of LVH in men versus women (Drazner 2011). Men tend to display eccentric remodeling of the left ventricle with concomitant myocyte dropout yet maintain chamber diameter, while women develop concentric hypertrophy and increased wall thickness (Krumholz et al. 1993). Women also have an increased prevalence of heart failure with preserved ejection fraction (HEFpEF), a malady that is at least partially attributed to a reduction in ventricular systolic and diastolic compliance (Kass 2002). HEFpEF is caused by increased ventricular and arterial stiffness which impact blood pressure lability, cardiovascular reserve capability, and coronary perfusion (Shibata et al. 2011). This pathological augmentation of ventricular elastance occurs more drastically in women during aging (Redfield et al. 2005), and is associated with large artery stiffening (Kass 2005). Although ElvI increased with exercise training in the women in our study, it is unlikely that this represents the pathological trajectory described above.

The increase in ElvI without the added stress of increased driving pressure suggests an advantage conferred by training in young, but not older, women. The attenuation in ESP may be due to a decrease in oxidative stress that improves nitric oxide (NO) signaling. Along these lines, Moreau et al. found that providing supplemental tetrahydrabiopterin (BH_4_, an essential co-factor for eNOS production of NO that is uncoupled by oxidative stress) rescued endothelial function in older, post-menopausal women (Moreau et al. 2012). Alternative explanations include improved β_2_-adrenergic responsiveness and resultant NO production or large artery remodeling, although the specific mechanism warrants further investigation. Importantly, aging, especially sedentary aging, is associated with augmented oxidative stress due in part to loss of β_2_-adrenergic responsiveness in women and uncoupling of BH_4_ (Moreau et al. 2005; Hart et al. 2011, 2012). Thus, the lack of improvement in LV performance in older women may be due to an inability to produce NO in the myocardium itself or in the proximal vessels to unload the heart that is at least partially attributable to a loss of estrogen.

Augmentation index is often greater in women, even when other factors affecting AIx, such as height, are controlled (Gatzka et al. 2001). Early wave reflection due to stiffer arteries or higher total peripheral resistance may cause AIx to become larger and, hence, increase ventricular afterload. The need for increased ventricular work to offset the contribution of reflected waves to ventricular afterload may explain the higher *E*_w_, EaI and ElvI in women both before and after training despite lower arterial pressure compared to men. Importantly, the relationship between peak myocardial wall stress and late-systolic wall stress depends on systemic vascular resistance, underscoring the importance of the microvasculature in mediating cardiac effort (Chirinos et al. 2012). There are also sex differences in the development of end-systolic myocardial wall stress that are dependent on the magnitude of wave reflection (Chirinos et al. 2012). Thus, strategies that reduce wave reflection such as the prevention of large artery stiffening and increased nitric oxide bioavailability may be important mediators of Ea and Elv, affecting cardiovascular reserve in both younger and older adults, especially in women.

Prior to the training intervention, the mean VO_2__peak_ score for women was at the lowest end of the “fair” fitness category, according to guidelines for cardiovascular fitness established by the American College of Sports Medicine (ACSM). After training, the average VO_2 peak_ score improved significantly (*p* < 0.05) but remained in the “fair” category. The men’s pre- and post-training VO_2 peak_ score followed the same pattern, and there was no correlation between the change in VO_2 peak_ value and the change in elastance values (*p* > 0.05 for all). Furthermore, the improvement in VO_2peak_ was 13 % for both men and women, suggesting that the training program produced similar improvements in both men and women. For this reason, sex differences in the degree of fitness improvement (assessed by VO_2 peak_) are unlikely to have played a role in the divergent ventricular elastance response between the sexes.

Previous work shows that left ventricular elastance and pressure–volume relationships in master’s athletes are similar to those of young controls, indicating a substantial effect of life-long training on ventricular and arterial compliance (Arbab-Zadeh et al. 2004; Shibata et al. 2008). However, others found that a year-long training intervention did little to improve ventricular and vascular coupling in older adults (Fujimoto et al. 2010), or improved coupling in older men (Rinder et al. 1999). Unlike older women (Spina et al. 1993), younger women are capable of improving ventricular performance. These findings highlight the necessity of beginning exercise training while young, especially in women, to maximize the benefits associated with cardiovascular exercise. This indicates that age at onset of training may be a crucial determinant of the potential for exercise training to offer protection by mediating LV adaptations, particularly in women. Nevertheless, because we did not include older men or women in our current study, potential age differences in the training response need to be specifically evaluated in future studies.

### Limitations

We did not invasively measure ESP, but the derivation of ESP from the reconstructed aortic waveform has been validated and yields almost identical values compared to invasive measurements (Holland et al. 2008). We also assumed the intercept of the pressure–volume loop (V_0_) to be equal to zero. This has been determined to be a reasonable approximation, as this value is unable to be non-invasively obtained and Sunagawa et al. (1983) have reported that the actual value is negligible compared to ESV and EDV. Thus, we do not believe that our results would be altered even if V_0_ would have been directly determined. Although the use of Ea as a surrogate for net arterial load does not directly account for the contribution of augmented pressure waves, Kelly et al. found Ea to be a reasonable and accurate approximation of net arterial load throughout a physiological range, even in hypertensive humans with AIx that far exceed those seen in the healthy population (Kelly et al. 1992). The men and women did have different SBP and MAP values throughout the study, which placed them in different BP categories: normotensive for women and pre-hypertensive for men. However, as there was no interaction between sexes for BP following training, so we believe these categorical differences minimally (if at all) impacted our main findings.

Our intervention controlled for frequency and length of training, but participants were able to select their own exercise intensity from the range recommended by the ACSM (60–90 % maximum HR). Although this did result in some variation in training intensity, we believe it replicates “real-world” exercise selection and improves applicability of the study to actual populations. Men and women completed a similar number of exercise sessions and the improvement in VO_2peak_ was similar between men and women, suggesting that the training stimulus was also similar between sexes.

We did not include a non-exercise control in the study. However, because the purpose of our study was to compare sex differences in the response to exercise training, it is unlikely that the control group would add additional insight to our findings.

## Conclusions

While women have higher EaI both before and after training, neither men nor women reduced EaI, but both groups reduced cPWV after 8 weeks of aerobic training. Women augmented ElvI post-training, leading to a reduction in their coupling ratio, while men retained their pre-training ElvI and EaI/ElvI values. Young men and women have divergent ElvI and coupling ratio responses to an 8-week endurance exercise intervention, and this may provide insight regarding sex-specific prevention of cardiovascular disease. This study also demonstrated that younger women may improve ventricular performance with exercise training and suggests that beginning training in young adulthood may be an important strategy for the prevention of cardiovascular disease, particularly in women.

## Abbreviations

AIx: Augmentation index
Ea: Arterial elastance
Ea/Elv: Ventricular–vascular coupling ratio
Elv: Ventricular elastance
ESP: End-systolic pressure
